
JOURNAL INFORMATION
==============================
NLM Title Abbreviation: ScientificWorldJournal
Journal ID: ScientificWorldJournal
NLM Journal ID: 101131163
Journal ID: TSWJ

The Scientific World Journal

ISSN: 2356-6140
EISSN: 1537-744X
Publisher: Wiley

ARTICLE INFORMATION
==============================
PMCID: PMC5823229
PMID: 19882086
DOI: 10.1100/tsw.2009.134
Article ID: 815749
Article version: 1
Subjects: Clinical Images

A Wii™-Related Clay-Shoveler's Fracture

Brown Christopher N. *
McKenna Patrick
Royal Berkshire Hospital, Reading, Berks, United Kingdom
*Christopher N. Brown: chrisnbrown79@googlemail.comAcademic Editor: Daniel Batlle

Publication date: 2009
Electronic publication date: 2009 Nov 1
Volume: 9
First page: 1190
Last page: 1191
Received 2009 Aug 14; Revised 2009 Oct 5; Accepted 2009 Oct 6
Copyright: Copyright © 2009 Christopher N. Brown and Patrick McKenna.
Copyright year: 2009
License: This is an open access article distributed under the Creative Commons Attribution License, which permits unrestricted use, distribution, and reproduction in any medium, provided the original work is properly cited.
License URL: https://creativecommons.org/licenses/by/3.0/

Keywords: clay-shoveler, WiiTM game console, fracture

==============================
Clinical Image
*TheScientificWorldJOURNAL* (2009) 9, 1190–1191
ISSN 1537-744X; DOI 10.1100/tsw.2009.134

TheScientificWorldJOURNAL
www.thescientificworld.com

### A Wii™-Related Clay-Shoveler's Fracture

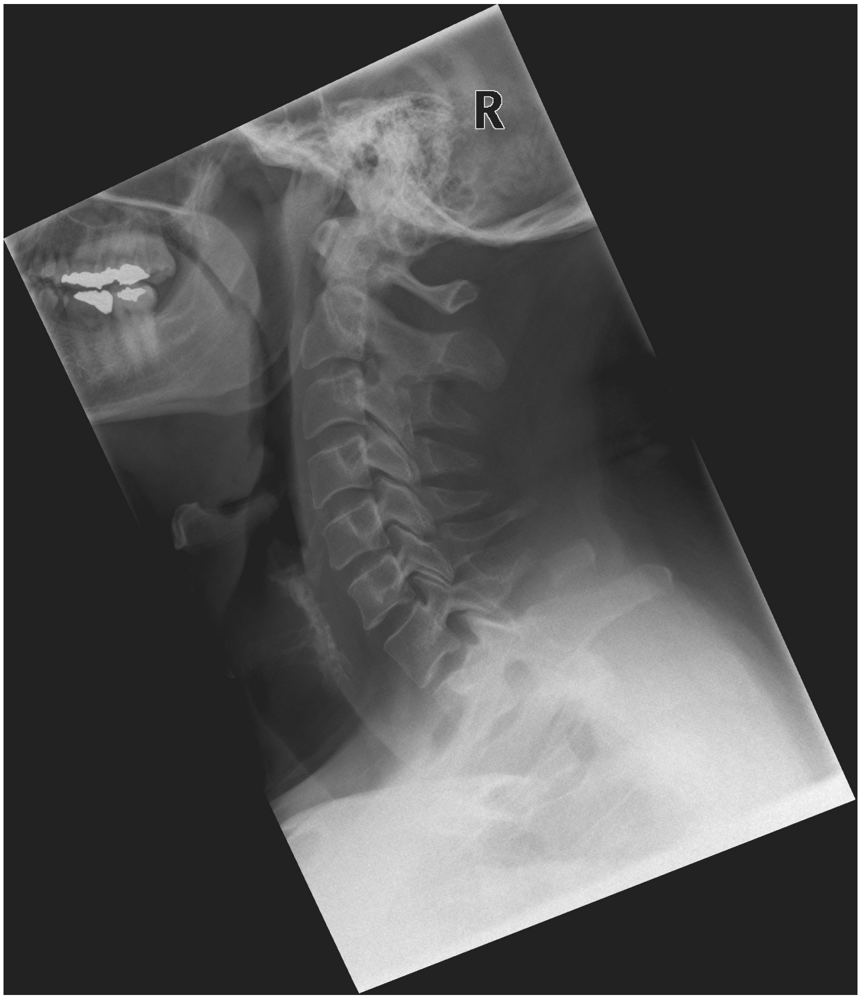

Christopher N. Brown* and Patrick McKenna
*Royal Berkshire Hospital, Reading, Berks, U.K.*

**E-mail**: chrisnbrown79@googlemail.com

*Received August 14, 2009; Revised October 5, 2009; Accepted October 6, 2009; Published November 1, 2009*

**KEYWORDS:** clay-shoveler, Wii™ game console, fracture

Wii™ is a registered trademark of Nintendo®.
*Corresponding author.
©2009 with author.
Published by TheScientificWorld; www.thescientificworld.com

Clay-shoveler's fractures are fatigue fractures of lower cervical or upper thoracic vertebral spinous processes. They are stable and normally managed conservatively, but occasionally require surgery for pain relief[1]. There are a number of reports of injuries, including fractures, which have been sustained during activities using game consoles[2]. To our knowledge, this is the first published report of a clay-shoveler's fracture attributable to using a Wii™ games console.

A 38-year-old man presented to the accident and emergency department complaining of severe neck pain. The patient reported that this injury had started immediately after swinging his Wii™ game console control during a rather vigorous game with his son. No other possible cause could be established and he was not found to have any neurological abnormalities on examination. He had previously suffered from very mild posterior cervical pain following a road traffic accident 6 years previously. X-rays demonstrated an acute fracture of the C7 spinous process. He was treated with immobilization in a soft collar followed by physiotherapy. Three months later, his posterior neck pain persisted and he therefore elected to have the fracture fragment excised. His symptoms resolved.

### REFERENCES

1. Hirsh, L.F., Duarte, L.E., Wolfson, E.A., and Gerhard, W. (1991) Isolated symptomatic cervical spinous process fracture requiring surgery. *J. Neurosurg*. **75(1),** 131–133.
2. Sparks, D., Chase, D., and Coughlin, L. (2009) Wii have a problem: a review of self-reported Wii related injuries. *Inform. Prim. Care* **17(1),** 55–57.

**This article should be cited as follows:**

Brown, C.N. and McKenna, P. (2009) A Wii™-related clay-shoveler's fracture. *TheScientificWorldJOURNAL* **9**, 1190–1191. DOI 10.1100/tsw.2009.134.
